# Supplementary material for: Phylogenetic clustering and rarity imply risk of local species extinction in prospective deep-sea mining areas of the Clarion–Clipperton Fracture Zone
Source: Proc Biol Sci. 2020 Apr 1;287(1924):20192666. doi: 10.1098/rspb.2019.2666 (PMC7209057; doi:10.1098/rspb.2019.2666)
Supplement: Supplementary Material [file rspb20192666supp1.docx]

Electronic Supplementary Material

Title: Phylogenetic clustering and rarity imply high risk of local species extinction in prospective deep-sea mining areas of the Clarion-Clipperton Zone

Lara Macheriotou^1^, Annelien Rigaux^1^, Sofie Derycke^1, 2^, Ann Vanreusel^1^

^1^Marine Biology Research Group, Department of Biology, Ghent University, Krijgslaan 281 7 building S8, 9000, Ghent, Belgium

^2^Aquatic Environment and Quality, Institute for Agricultural and Fisheries Research (ILVO), Ankerstraat 1, 8400 Oostende, Belgium

Table 1: Longitude, latitude average depth and number of respective replicates of the six sampling Areas (BGR.PA, BGR.RA: Bundesanstalt für Geowissenschaften und Rohstoffe Prospective Area and Reference Area respectively, IOM.C: InterOcean Metal-Control, GSR: Global Seabed Resources, IFREMER: Institut Français de Recherche pour l'Exploitation de la Mer, APEI3: Area of Particular Environmental Interest #3). Replicates of each Area were retrieved from separate mutlicorer deployments.

| Area | Longitude | Latitude | Depth (m) | Replicates |
| --- | --- | --- | --- | --- |
| BGR.PA | 117° 03" W | 11° 50" N | 4132 | 5 |
| BGR.RA | 117° 32" W | 11° 49" N | 4351 | 4 |
| IOM.C | 119° 39" W | 11° 04" N | 4437 | 2 |
| GSR | 123° 15" W | 13° 51" N | 4513 | 4 |
| IFREMER | 130° 08" W | 14° 03" N | 4949 | 5 |
| APEI3 | 128° 21" W | 18° 47" N | 4839 | 3 |


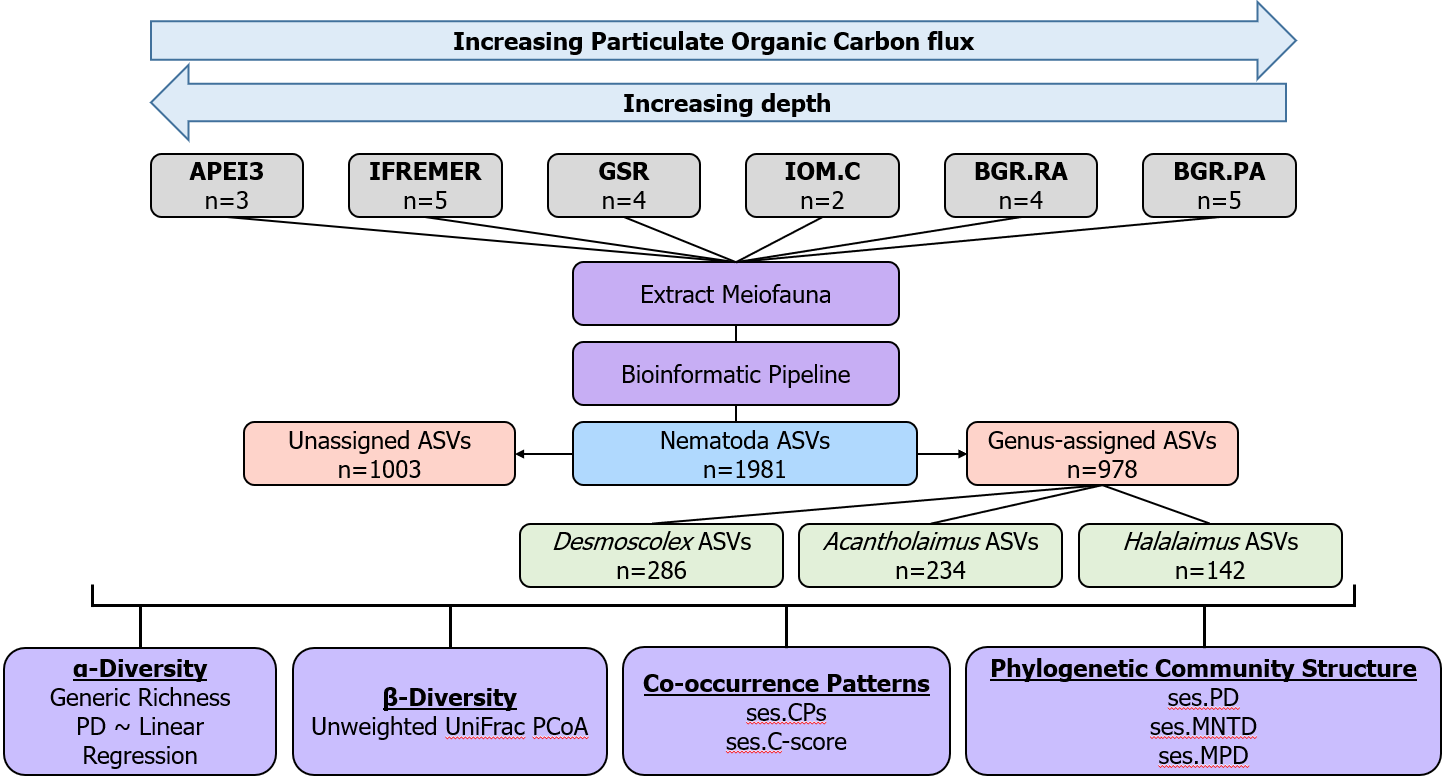
Figure 1S: Sampling design: Six Areas were sampled on an East to West transect along a Particulate Organic Carbon and depth gradient (grey boxes with respective replicates). Meiofauna was extracted from each replicate, to which the bioinformatic pipeline was applied, resulting in a total of 1981 Nematoda ASVs. Of these, 1003 were Unassigned at genus-level, 978 were Genus-assigned. From the Genus-assigned ASVs, 286, 234, and 142 were assigned to the genera *Desmoscolex*, *Acantholaimus* and *Halalaimus*, respectively.

## DNA extraction, HTS Library Preparation

Genomic DNA was extracted by adding 6 µL proteinase-K [10mg/mL] and centrifuged 5 min at 14000 rpm (Room Temperature, RT). The pellet was ground, bead-beaten for 2 min at 30 cycles/second and incubated at 60°C for 1 hour. Ammoniumacetate (250 µL, 7.5 M) was added and tubes centrifuged for 10 min at 14000 rpm (RT). The supernatant (750 µL) was transferred into a new sterile tube; 750 µL of cold 80% isopropanol was added, mixed, incubated for 30 min at RT, and centrifuged for 15 min at 14000 rpm (4°C). The supernatant was removed, 1 mL washing buffer (76% EtOH 10mM ammonium acetate) was added, tubes were incubated for 30 min on ice (to remove salts) and centrifuged for 5 min at 14000 rpm (4°C). Finally, the supernatant was removed, and 20 µL of sterile water was added.

The 18S (V1-V2 region) ribosomal locus was amplified using the primers SSU_F_04-SSU/22_R (GCTTGTCTCAAAGATTAAGCC, TCCAAGGAAGGCAGCAGGC respectively) which were constructed with Illumina overhang adapters as described in “16S Metagenomic Sequencing Library Preparation”. Each sample was amplified in triplicate with the following PCR conditions: 95°C 2 min, 30x(95 °C 1 min, 57 °C 45 sec, 72 °C 1 min), 72 °C 10 min. The mix consisted of 8.4 μL PCR-grade H_2_O, 4 µL Phusion Buffer, 4 µL Dye, 0.4 µL dNTP [10 mM], 1 µL forward and reverse primer [10 μM], 0.2 µL Phusion Hot Start II High Fidelity Polymerase (New England BioLabs, U.S.A.) and 1 µL DNA template (diluted 1/10). In the event of failed amplification, DNA templates were diluted 1/50 in PCR-grade H_2_O and/or 2 µL template were used. PCR products were run on a 1% agarose electrophoresis gel, triplicates were pooled, purified using Agencourt AMPure XP beads and run on Bioanalyzer 2100 High Sensitivity to confirm length and size distribution of the PCR fragments. Library indexing was completed using the FC131-1002 NexteraXT Index Kit (Illumina, U.S.A.) and Kapa High Fidelity PCR kit (Kapa Biosystems, U.S.A.). The mix consisted of 11.25 μL PCR-grade H_2_O, 5 µL Buffer, 0.75 µL dNTP [10 mM], 2.5 µL Index1 and Index2, 0.5 µL Kapa Hot Start High Fidelity Polymerase and 2.5 µL PCR product. These were then purified using Agencourt AMPure XP beads and 11 randomly chosen samples were run on Bioanalyzer 2100 High Sensitivity to confirm successful indexing. DNA was quantified using Qubit® dsDNA High Sensitivity Assay Kit in all samples for pooling. Finally, biological replicates were distributed over three pooled libraries and sequenced at Edinburgh Genomics on three Illumina MiSeq-v3 2x300bp paired-end read runs (<https://genomics.ed.ac.uk/>).


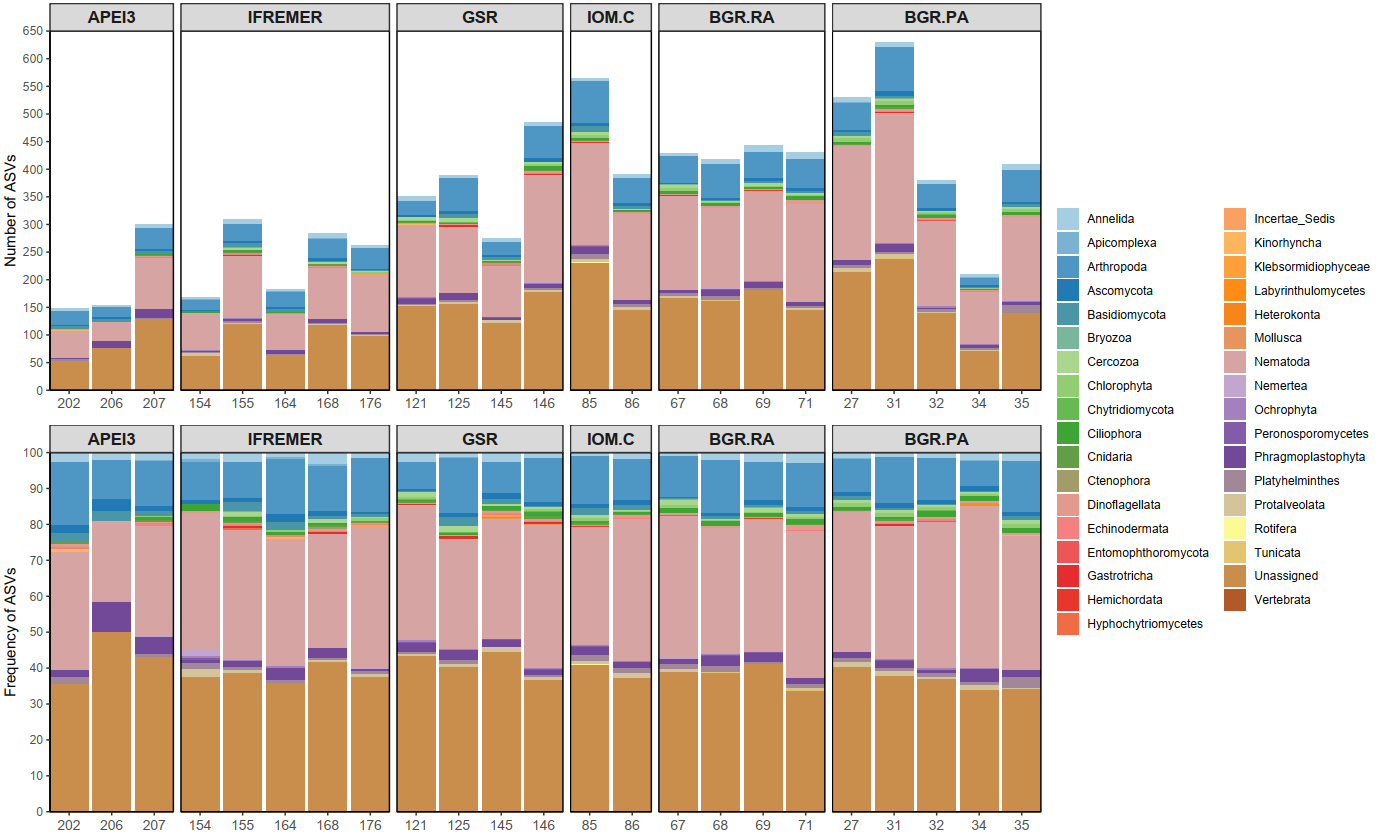
Figure 2S: Phylum-level taxonomic assignments of rarefied ASVs in the BGR.PA, BGR.RA, IOM.C, GSR, IFREMER and APEI Areas.


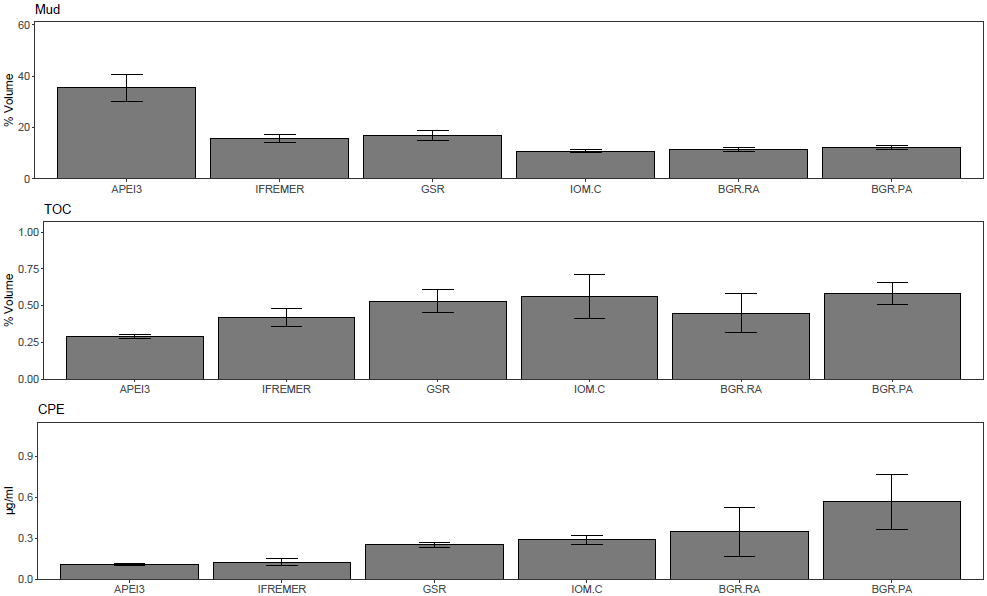
 Figure 3S: Environmental variables %Total Organic Carbon (%TOC), %Mud () and Chloroplastic Pigments Equivalent (CPE, μg/mL) in the BGR.PA, BGR.RA, IOM.C, GSR, IFREMER and APEI3 Areas. Error bars represent standard deviation.


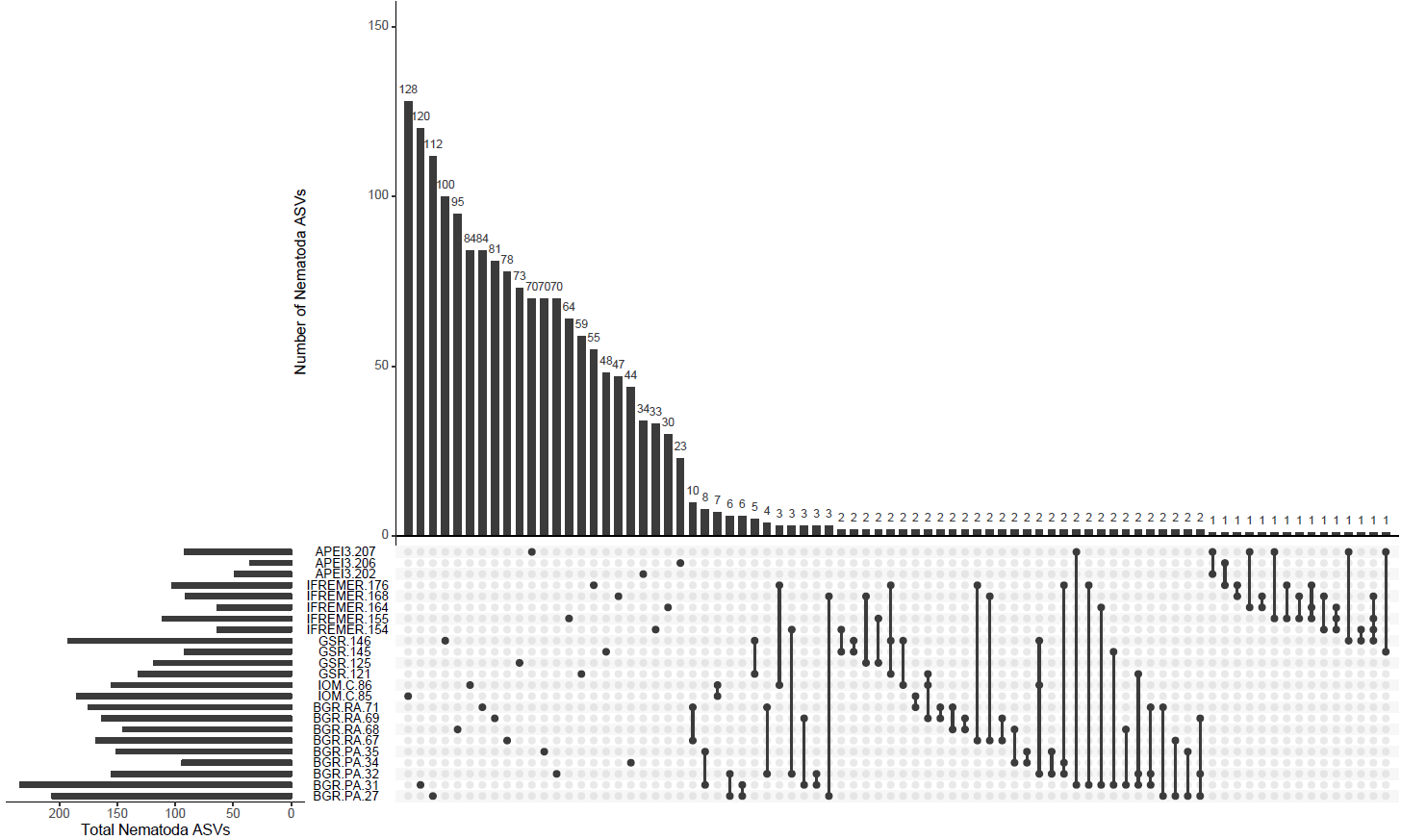


Figure 4S: Unique and shared Nematoda ASVs by replicate core. Main panel: number above bars indicate number of Nematoda ASVs found in Areas marked by filled circles; a single filled circle/column signifies unique ASVs (i.e. not shared with another sampling location). Bottom left graph: total number of Nematoda ASVs in the BGR.PA, BGR.RA, IOM.C, GSR, IFREMER and APEI3 Areas.


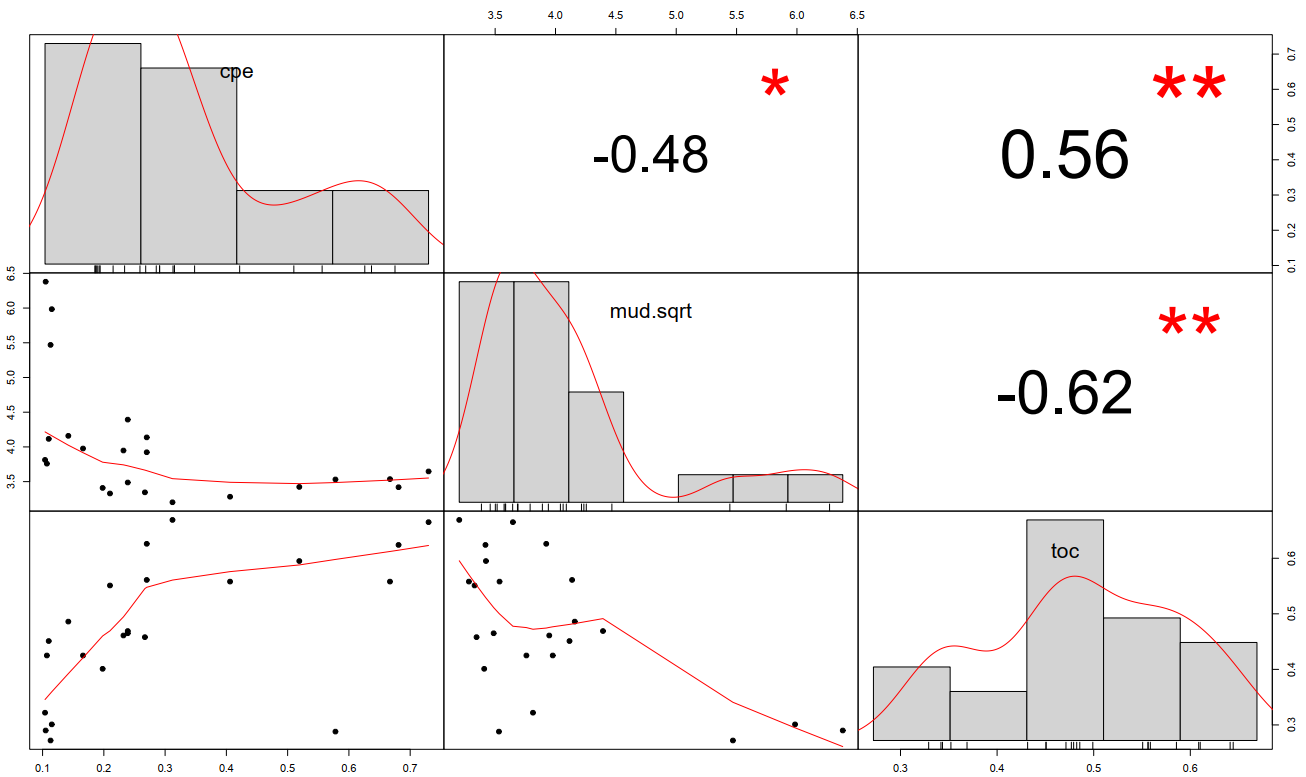


Figure 5S: Correlation chart for environmental variables %Total Organic Carbon (toc), square-root %Mud (mud.sqrt, <4 µm) and Chloroplastic Pigment Equivalent (CPE, Σ chlorophyll-a + phaeopigments). The distribution of each variable is shown on the diagonal; bivariate scatter plots with a fitted line are displayed on the bottom of the diagonal; the value of the (Pearson) correlation plus the significance level as stars are displayed on top of the diagonal [p-values (0, 0.001, 0.01, 0.05, 0.1, 1) <=> symbols (“***”, “**”, “*”, “.”, " “)].

Table 2: Average (Avg.) and standard deviation (St. dev.) for generic richness of Nematoda ASVs the BGR.PA, BGR.RA, IOM.C, GSR, IFREMER and APEI3 Areas.

| Area | Avg. | St. dev. |
| --- | --- | --- |
| APEI3 | 9.000 | 3.606 |
| IFREMER | 12.800 | 3.834 |
| GSR | 15.750 | 3.304 |
| IOM.C | 19.500 | 0.707 |
| BGR.RA | 20.250 | 1.500 |
| BGR.PA | 20.600 | 2.074 |

Table 3: Results of statistical testing (p-value) for data normality (Shapiro-Wilk, S-W), homoscedasticity (Levene) and Analysis of Variance (ANOVA) for generic richness of Nematoda ASVs in the BGR.PA, BGR.RA, IOM.C, GSR, IFREMER and APEI3 Areas. Significant values indicated in grey (α=0.05). Note: Normality assumption could not be tested in IOM.C data due to insufficient replication (n=2).

| Area | S-W | Levene | ANOVA |
| --- | --- | --- | --- |
| APEI3 | 0.537 | 0.415 | 0.000 |
| IFREMER | 0.961 |  |  |
| GSR | 0.513 |  |  |
| IOM.C | --- |  |  |
| BGR.RA | 0.224 |  |  |
| BGR.PA | 0.023 |  |  |

| Groups | p-Tukey |
| --- | --- |
| IFREMER-APEI3 | 0.491 |
| GSR-APEI3 | 0.066 |
| IOM.C-APEI3 | 0.010 |
| BGR.RA-APEI3 | 0.001 |
| BGR.PA-APEI3 | 0.000 |
| GSR-IFREMER | 0.656 |
| IOM.C-IFREMER | 0.111 |
| BGR.RA-IFREMER | 0.014 |
| BGR.PA-IFREMER | 0.006 |
| IOM.C-GSR | 0.670 |
| BGR.RA-GSR | 0.287 |
| BGR.PA-GSR | 0.178 |
| BGR.RA-IOM.C | 1.000 |
| BGR.PA-IOM.C | 0.997 |
| BGR.PA-BGR.RA | 1.000 |

Table 4: Tukey's HSD (Honestly Significant Difference) test results (p-value) for pairwise comparisons for generic richness of Nematoda ASVS in the BGR.PA, BGR.RA, IOM.C, GSR, IFREMER and APEI3 Areas. Significant values indicated in grey (α=0.05).

Table 5: Average (Avg.) and standard deviation (St. Dev.) for Phylogenetic Diversity (PD) in the BGR.PA, BGR.RA, IOM.C, GSR, IFREMER and APEI3 Areas.

| Level | Area | PD | |
| --- | --- | --- | --- |
|  |  | Avg. | St. dev. |
| Nematoda | APEI3 | 3.746 | 1.539 |
|  | IFREMER | 5.395 | 1.041 |
|  | GSR | 7.187 | 1.271 |
|  | IOM.C | 8.481 | 0.898 |
|  | BGR.RA | 7.813 | 0.241 |
|  | BGR.PA | 7.953 | 1.671 |
| Genus-assigned | APEI3 | 1.824 | 0.442 |
|  | IFREMER | 2.562 | 0.503 |
|  | GSR | 3.241 | 0.471 |
|  | IOM.C | 4.130 | 0.167 |
|  | BGR.RA | 4.069 | 0.394 |
|  | BGR.PA | 4.054 | 0.732 |
| Unassigned | APEI3 | 2.759 | 1.206 |
|  | IFREMER | 4.059 | 0.856 |
|  | GSR | 5.527 | 1.035 |
|  | IOM.C | 5.997 | 0.732 |
|  | BGR.RA | 5.381 | 0.335 |
|  | BGR.PA | 5.669 | 1.243 |
| *Acantholaimus* | APEI3 | 0.210 | 0.056 |
|  | IFREMER | 0.216 | 0.053 |
|  | GSR | 0.251 | 0.052 |
|  | IOM.C | 0.403 | 0.214 |
|  | BGR.RA | 0.298 | 0.082 |
|  | BGR.PA | 0.305 | 0.105 |
| *Desmoscolex* | APEI3 | 0.439 | 0.083 |
|  | IFREMER | 0.517 | 0.137 |
|  | GSR | 0.661 | 0.135 |
|  | IOM.C | 1.021 | 0.125 |
|  | BGR.RA | 0.859 | 0.178 |
|  | BGR.PA | 0.902 | 0.298 |
| *Halalaimus* | APEI3 | 0.250 | 0.055 |
|  | IFREMER | 0.331 | 0.062 |
|  | GSR | 0.333 | 0.028 |
|  | IOM.C | 0.527 | 0.065 |
|  | BGR.RA | 0.429 | 0.043 |
|  | BGR.PA | 0.392 | 0.163 |

Table 6: Results of statistical testing (p-value) for data normality (Shapiro-Wilk, S-W), homoscedasticity (Levene) and Analysis of Variance (ANOVA) for Phylogenetic Diversity (PD) within Nematoda, Genus-assigned, Unassigned, *Acantholaimus*, *Desmoscolex* and *Halalaimus* ASVs in each Area. Non-parametric equivalents (PERMDISP, PERMANOVA) where used when necessary (i.e. Shapiro-Wilk/Levenes p<0.05, n<3). Significant values indicated in grey (α=0.05).

| Level | Area | PD | | |
| --- | --- | --- | --- | --- |
|  |  | Shapiro-Wilk | Levene | p-ANOVA |
| All Nematoda | APEI3 | 0.4877 | 0.5467 | 0.0039 |
|  | IFREMER | 0.0576 |  |  |
|  | GSR | 0.2651 |  |  |
|  | IOM.C | --- |  |  |
|  | BGR.RA | 0.1883 |  |  |
|  | BGR.PA | 0.3947 |  |  |
| Genus-assigned | APEI3 | 0.9863 | 0.5861 | 0.0003 |
|  | IFREMER | 0.9400 |  |  |
|  | GSR | 0.4886 |  |  |
|  | IOM.C | --- |  |  |
|  | BGR.RA | 0.2669 |  |  |
|  | BGR.PA | 0.5535 |  |  |
| Unassigned | APEI3 | 0.3687 | 0.6745 | 0.0145 |
|  | IFREMER | 0.6335 |  |  |
|  | GSR | 0.2112 |  |  |
|  | IOM.C | --- |  |  |
|  | BGR.RA | 0.3154 |  |  |
|  | BGR.PA | 0.1910 |  |  |
| *Acantholaimus* | APEI3 | 0.7264 | 0.0386 | PERMDISP |
|  | IFREMER | 0.1392 |  | 0.5386 |
|  | GSR | 0.4743 |  | PERMANOVA |
|  | IOM.C | --- |  | 0.579 |
|  | BGR.RA | 0.6639 |  |  |
|  | BGR.PA | 0.0738 |  |  |
| *Desmoscolex* | APEI3 | --- | 0.7190 | 0.0320 |
|  | IFREMER | 0.6702 |  |  |
|  | GSR | 0.6354 |  |  |
|  | IOM.C | --- |  |  |
|  | BGR.RA | 0.5322 |  |  |
|  | BGR.PA | 0.6122 |  |  |
| *Halalaimus* | APEI3 | 0.7055 | 0.0585 | 0.0911 |
|  | IFREMER | 0.8784 |  |  |
|  | GSR | 0.4371 |  |  |
|  | IOM.C | --- |  |  |
|  | BGR.RA | 0.5431 |  |  |
|  | BGR.PA | 0.4102 |  |  |

Table 7: Tukey's HSD (Honestly Significant Difference) test results (adjusted p-value) for pairwise comparisons of Phylogenetic Diversity (PD) for Nematoda, Genus-assigned, Unassigned and *Desmoscolex* ASVs in the BGR.PA, BGR.RA, IOM.C, GSR, IFREMER and APEI3 Areas. Significant values indicated in grey (α=0.05).

| Groups | Nematoda | Genus-assigned | Unassigned | *Desmoscolex* |
| --- | --- | --- | --- | --- |
| IFREMER-APEI3 | 0.623 | 0.557 | 0.621 | 0.998 |
| GSR-APEI3 | 0.055 | 0.061 | 0.048 | 0.848 |
| IOM.C-APEI3 | 0.021 | 0.006 | 0.055 | 0.146 |
| BGR.RA-APEI3 | 0.018 | 0.002 | 0.067 | 0.293 |
| BGR.PA-APEI3 | 0.010 | 0.001 | 0.026 | 0.181 |
| GSR-IFREMER | 0.453 | 0.555 | 0.412 | 0.920 |
| IOM.C-IFREMER | 0.156 | 0.057 | 0.353 | 0.124 |
| BGR.RA-IFREMER | 0.173 | 0.017 | 0.520 | 0.248 |
| BGR.PA-IFREMER | 0.101 | 0.011 | 0.263 | 0.118 |
| IOM.C-GSR | 0.896 | 0.541 | 0.996 | 0.449 |
| BGR.RA-GSR | 0.988 | 0.404 | 1.000 | 0.800 |
| BGR.PA-GSR | 0.964 | 0.369 | 1.000 | 0.597 |
| BGR.RA-IOM.C | 0.994 | 1.000 | 0.987 | 0.954 |
| BGR.PA-IOM.C | 0.998 | 1.000 | 0.999 | 0.986 |
| BGR.PA-BGR.RA | 1.000 | 1.000 | 0.999 | 1.000 |

Table 8: Partial and global p-values and adjusted R-squared values for linear regression analyses of PD for Nematoda, Unassigned, Genus-assigned, *Acantholaimus*, *Desmoscolex*, *Halalaimus* ASVs and environmental variables %Total Organic Carbon (TOC), square-root %Mud (Mud.sqrt, <4 µm) and Chloroplastic Pigment Equivalent (CPE, Σ chlorophyll-a + phaeopigments).

| PD | CPE | TOC | Mud.sqrt | CPE:TOC | Global p-value | Adj. R^2^ |
| --- | --- | --- | --- | --- | --- | --- |
|  | p-value | | | | | |
| Nematoda | 0.0090 | 0.0040 | - | 0.0207 | 0.0015 | 0.4764 |
| Unassigned | - | 0.0020 | - | - | 0.0025 | 0.3293 |
| Genus-assigned | 0.0210 | - | 0.0045 | - | 0.0001 | 0.5720 |
| *Acantholaimus* | - | - | - | - | - | - |
| *Desmoscolex* | - | - | - | - | - | - |
| *Halalaimus* | - | - | 0.0038 | - | 0.0038 | 0.3030 |

Table 9: Observed, simulated (mean), lower/upper-tail p-values (2-tailed t-test) and standardized effect size (SES) for checkerboard pairs (CPs) and C-score of Nematoda, *Acantholaimus*, *Desmoscolex* and *Halalaimus* ASVs within CCFZ (pooled/unpooled) and BGR.PA, BGR.RA, IOM.C, GSR, IFREMER and APEI3. Significant values indicated in grey (α=0.05).

| Checkerboard pairs | **Nematoda** | | | | | | | |
| --- | --- | --- | --- | --- | --- | --- | --- | --- |
|  | Regional | | Local | | | | | |
| **CPs** | CCFZ.Replicates | CCFZ.Areas | APEI3 | IFREMER | GSR | IOM.C | BGR.RA | BGR.PA |
| Observed | 1755971 | 1399727 | 8413 | 49488 | 61554 | 19375 | 93978 | 138078 |
| Simulated_mean | 1780635.000 | 1465388.000 | 9239.700 | 50288.000 | 63823.000 | 19531.000 | 93722.000 | 143483.000 |
| Lower-tail P | 0.000 | 0.000 | 0.000 | 0.000 | 0.000 | 0.084 | 0.999 | 0.000 |
| Upper-tail P | 1.000 | 1.000 | 1.000 | 1.000 | 1.000 | 0.935 | 0.001 | 1.000 |
| SES | -65.544 | -119.180 | -14.818 | -7.391 | -16.543 | -1.588 | 1.541 | -28.777 |
| %CPs_obs | 89.536 | 71.371 | 58.566 | 72.103 | 62.589 | 40.453 | 64.816 | 65.665 |
| %CPs_sim | 90.794 | 74.719 | 64.321 | 73.269 | 64.896 | 40.779 | 64.640 | 68.236 |
| **C-score** |  | | | | | | | |
| Observed | 1.698 | 1399727.000 | 0.600 | 0.856 | 0.749 | 0.405 | 0.789 | 0.853 |
| Simulated_mean | 1.762 | 1465387.000 | 0.659 | 0.870 | 0.789 | 0.408 | 0.789 | 0.892 |
| Lower-tail P | 0.000 | 0.000 | 0.000 | 0.001 | 0.000 | 0.080 | 0.413 | 0.000 |
| Upper-tail P | 1.000 | 1.000 | 1.000 | 0.999 | 1.000 | 0.936 | 0.587 | 1.000 |
| SES | -65.471 | -119.700 | -14.475 | -5.798 | -18.345 | -1.585 | 0.024 | -24.550 |
|  | ***Acantholaimus*** | | | | | | | |
|  | Regional | | Local | | | | | |
| **CPs** | CCFZ.Replicates | CCFZ.Areas | APEI3 | IFREMER | GSR | IOM.C | BGR.RA | BGR.PA |
| Observed | 23293 | 18631 | 248 | 886 | 749 | 286 | 1267 | 1622 |
| Simulated_mean | 23655.000 | 19151.000 | 270.630 | 912.620 | 785.960 | 332.840 | 1261.400 | 1769.500 |
| Lower-tail P | 0.000 | 0.000 | 0.038 | 0.070 | 0.037 | 0.023 | 0.531 | 0.000 |
| Upper-tail P | 1.000 | 1.000 | 0.968 | 0.935 | 0.966 | 0.992 | 0.493 | 1.000 |
| SES | -7.658 | -7.996 | -2.327 | -1.665 | -2.253 | -3.526 | 0.287 | -6.575 |
| %CPs_obs | 85.444 | 68.343 | 53.333 | 61.915 | 50.438 | 33.217 | 60.913 | 52.645 |
| %CPs_sim | 86.772 | 70.251 | 58.200 | 63.775 | 52.927 | 38.657 | 60.644 | 57.433 |
| **C-score** |  |  |  |  |  |  |  |  |
| Observed | 2.198 | 0.933 | 0.581 | 0.864 | 0.735 | 0.332 | 0.788 | 0.809 |
| Simulated_mean | 2.280 | 0.985 | 0.623 | 0.906 | 0.777 | 0.387 | 0.773 | 0.882 |
| Lower-tail P | 0.000 | 0.000 | 0.070 | 0.044 | 0.044 | 0.020 | 0.887 | 0.001 |
| Upper-tail P | 1.000 | 1.000 | 0.942 | 0.958 | 0.958 | 0.992 | 0.130 | 0.999 |
| SES | -6.407 | -9.505 | -1.727 | -2.047 | -2.088 | -3.529 | 0.955 | -4.811 |
|  | ***Desmoscolex*** | | | | | | | |
|  | Regional | | Local | | | | | |
| **CPs** | CCFZ.Replicates | CCFZ.Areas | APEI3 | IFREMER | GSR | IOM.C | BGR.RA | BGR.PA |
| Observed | 36936 | 29721 | 60 | 844 | 1070 | 513 | 1803 | 3099 |
| Simulated_mean | 37584.000 | 31319.000 | 80.075 | 836.860 | 1126.800 | 517.230 | 1786.000 | 3220.700 |
| Lower-tail P | 0.000 | 0.000 | 0.008 | 0.660 | 0.013 | 0.308 | 0.773 | 0.002 |
| Upper-tail P | 1.000 | 1.000 | 0.994 | 0.378 | 0.988 | 0.814 | 0.250 | 0.998 |
| SES | -12.524 | -19.536 | -3.947 | 0.518 | -3.234 | -0.255 | 0.754 | -4.251 |
| %CPs_obs | 90.629 | 72.926 | 50.000 | 78.076 | 67.043 | 41.878 | 66.753 | 67.961 |
| %CPs_sim | 92.219 | 76.847 | 66.729 | 77.415 | 70.602 | 42.223 | 66.124 | 70.629 |
| **C-score** |  | | | | | | | |
| Observed | 1.526 | 0.911 | 0.500 | 0.841 | 0.719 | 0.419 | 0.816 | 0.880 |
| Simulated_mean | 1.587 | 0.986 | 0.666 | 0.834 | 0.776 | 0.422 | 0.812 | 0.930 |
| Lower-tail P | 0.000 | 0.000 | 0.007 | 0.597 | 0.004 | 0.306 | 0.530 | 0.001 |
| Upper-tail P | 1.000 | 1.000 | 0.994 | 0.424 | 0.996 | 0.815 | 0.483 | 0.999 |
| SES | -13.717 | -21.331 | -3.811 | 0.420 | -4.011 | -0.271 | 0.283 | -4.552 |
|  | ***Halalaimus*** | | | | | | | |
|  | Regional | | Local | | | | | |
| **CPs** | CCFZ.Replicates | CCFZ.Areas | APEI3 | IFREMER | GSR | IOM.C | BGR.RA | BGR.PA |
| Observed | 8823 | 6823 | 157 | 396 | 337 | 130 | 533 | 657 |
| Simulated_mean | 8838.300 | 6917.000 | 154.090 | 394.640 | 325.950 | 126.420 | 511.350 | 680.900 |
| Lower-tail P | 0.287 | 0.031 | 0.637 | 0.480 | 0.986 | 0.682 | 0.999 | 0.066 |
| Upper-tail P | 0.724 | 0.970 | 0.485 | 0.560 | 0.048 | 0.592 | 0.001 | 0.939 |
| SES | -0.515 | -2.280 | 0.409 | 0.134 | 1.145 | 0.453 | 1.680 | -1.739 |
| %CPs_obs | 88.133 | 68.155 | 67.965 | 70.588 | 67.944 | 37.037 | 65.000 | 66.364 |
| %CPs_sim | 88.286 | 69.094 | 66.706 | 70.346 | 65.716 | 36.017 | 62.360 | 68.778 |
| **C-score** |  | | | | | | | |
| Observed | 2.307 | 1.059 | 0.680 | 0.948 | 0.776 | 0.370 | 0.916 | 0.971 |
| Simulated_mean | 2.321 | 1.092 | 0.667 | 0.947 | 0.749 | 0.360 | 0.877 | 1.007 |
| Lower-tail P | 0.165 | 0.005 | 0.636 | 0.443 | 0.951 | 0.680 | 0.993 | 0.091 |
| Upper-tail P | 0.836 | 0.995 | 0.488 | 0.582 | 0.090 | 0.591 | 0.009 | 0.913 |
| SES | -0.955 | -3.462 | 0.411 | 0.052 | 1.073 | 0.444 | 1.473 | -1.457 |

Table 10: Average (Avg.) and standard deviation (St. Dev.) for standardized effect size of PD (ses.PD), unweighted Mean Pairwise Distance (ses.MPD) and unweighted Mean Nearest Taxon Distance (ses.MNTD) for Nematoda, Genus-assigned, Unassigned, *Acantholaimus*, *Desmoscolex* and *Halalaimus* ASVs in the BGR.PA, BGR.RA, IOM.C, GSR, IFREMER and APEI3 Areas.

| Level | Area | ses.PD | | ses.MPD | | ses.MNTD | |
| --- | --- | --- | --- | --- | --- | --- | --- |
|  |  | Avg. | St. dev. | Avg. | St. dev. | Avg. | St. dev. |
| Nematoda | APEI3 | -1.934 | 0.858 | -0.553 | 0.091 | -2.140 | 0.784 |
|  | IFREMER | -1.170 | 0.652 | -0.891 | 0.347 | -1.104 | 0.720 |
|  | GSR | -1.327 | 0.950 | -0.903 | 0.476 | -1.602 | 1.303 |
|  | IOM.C | -1.498 | 0.646 | -1.731 | 0.952 | -1.718 | 0.662 |
|  | BGR.RA | -2.241 | 0.462 | -1.831 | 0.634 | -2.377 | 0.552 |
|  | BGR.PA | -2.149 | 1.170 | -2.283 | 1.588 | -2.368 | 1.214 |
| Genus-assigned | APEI3 | -2.419 | 0.491 | -0.607 | 1.072 | -2.297 | 0.688 |
|  | IFREMER | -1.700 | 0.817 | -0.654 | 0.679 | -1.516 | 1.002 |
|  | GSR | -1.439 | 0.448 | -0.841 | 1.228 | -1.015 | 0.478 |
|  | IOM.C | -1.448 | 0.065 | -0.364 | 0.940 | -1.641 | 0.277 |
|  | BGR.RA | -1.083 | 1.360 | -0.457 | 1.106 | -1.531 | 1.114 |
|  | BGR.PA | -1.248 | 1.372 | -0.987 | 1.104 | -1.437 | 1.221 |
| Unassigned | APEI3 | -0.335 | 0.194 | -0.457 | 0.294 | -1.106 | 0.409 |
|  | IFREMER | 0.111 | 1.407 | -0.237 | 0.883 | -0.025 | 1.495 |
|  | GSR | -0.480 | 0.882 | -0.379 | 0.718 | -0.799 | 1.426 |
|  | IOM.C | -0.710 | 0.425 | -1.863 | 0.451 | -0.267 | 0.561 |
|  | BGR.RA | -1.805 | 0.159 | -1.630 | 0.808 | -1.834 | 0.198 |
|  | BGR.PA | -1.217 | 0.760 | -1.586 | 1.550 | -1.517 | 0.928 |
| *Acantholaimus* | APEI3 | -0.244 | 0.191 | -0.442 | 0.052 | -0.949 | 0.124 |
|  | IFREMER | -0.999 | 0.359 | -1.366 | 0.338 | -1.412 | 0.316 |
|  | GSR | -1.305 | 0.518 | -1.618 | 0.518 | -1.656 | 0.404 |
|  | IOM.C | 0.085 | 1.695 | -0.275 | 1.771 | -0.042 | 1.874 |
|  | BGR.RA | -0.911 | 0.824 | -1.036 | 0.581 | -1.283 | 0.808 |
|  | BGR.PA | -1.324 | 1.083 | -1.350 | 0.889 | -2.074 | 0.998 |
| *Desmoscolex* | APEI3 | 0.029 | 0.042 | -0.286 | 0.195 | -0.035 | 0.076 |
|  | IFREMER | -0.068 | 0.607 | -0.344 | 0.796 | -0.108 | 0.524 |
|  | GSR | -0.261 | 0.809 | -0.519 | 0.936 | -0.095 | 0.690 |
|  | IOM.C | -0.464 | 0.113 | -0.119 | 0.388 | -0.970 | 0.111 |
|  | BGR.RA | -0.523 | 0.990 | -0.871 | 1.029 | -0.340 | 1.063 |
|  | BGR.PA | -0.469 | 1.164 | -0.351 | 0.950 | -0.826 | 1.061 |
| *Halalaimus* | APEI3 | -0.892 | 0.560 | -1.034 | 0.304 | -0.918 | 0.421 |
|  | IFREMER | -0.227 | 0.702 | -0.330 | 0.742 | -0.213 | 0.713 |
|  | GSR | -0.640 | 0.454 | -0.657 | 0.686 | -0.568 | 0.575 |
|  | IOM.C | -0.003 | 0.392 | 0.152 | 0.422 | -0.318 | 0.450 |
|  | BGR.RA | -0.439 | 0.423 | -0.603 | 0.494 | -0.237 | 0.520 |
|  | BGR.PA | -0.432 | 0.829 | -0.223 | 0.869 | -0.476 | 0.870 |

Table 11: Statistical testing of normality (Shapiro-Wilk, S-W) and non-zero means (t-test) for standardised effect size of Phylogenetic Diversity (ses.PD), unweighted Mean Pairwise Distance (ses.MPD) and unweighted Mean Nearest Taxon Distance (ses.MNTD) for Nematoda, Genus-assigned, Unassigned, *Acantholaimus*, *Desmoscolex* and *Halalaimus* ASVs in the BGR.PA, BGR.RA, IOM.C, GSR, IFREMER and APEI3 Areas. Significant values indicated in grey (α=0.05).

| Level | Area | ses.PD | | | ses.MNTD | | | ses.MPD | | |
| --- | --- | --- | --- | --- | --- | --- | --- | --- | --- | --- |
|  |  | S-W | t.test | W | S-W | t.test | W | S-W | t.test | W |
| Nematoda | APEI3 | 0.592 | 0.086 |  | 0.922 | 0.061 |  | 0.531 | 0.013 |  |
|  | IFREMER | 0.064 | 0.023 |  | 0.065 | 0.037 |  | 0.263 | 0.007 |  |
|  | GSR | 0.686 | 0.094 |  | 0.712 | 0.123 |  | 0.492 | 0.046 |  |
|  | IOM.C |  |  | 0.500 |  |  | 0.500 |  |  | 0.500 |
|  | BGR.RA | 0.268 | 0.004 |  | 0.890 | 0.005 |  | 0.077 | 0.015 |  |
|  | BGR.PA | 0.647 | 0.021 |  | 0.875 | 0.018 |  | 0.747 | 0.045 |  |
| Genus-assigned | APEI3 | 0.508 | 0.020 |  | 0.974 | 0.042 |  | 0.714 | 0.507 |  |
|  | IFREMER | 0.125 | 0.014 |  | 0.425 | 0.039 |  | 0.064 | 0.126 |  |
|  | GSR | 0.280 | 0.011 |  | 0.825 | 0.035 |  | 0.725 | 0.321 |  |
|  | IOM.C |  |  | 0.500 |  |  | 0.500 |  |  | 1.000 |
|  | BGR.RA | 0.897 | 0.262 |  | 0.516 | 0.098 |  | 0.998 | 0.526 |  |
|  | BGR.PA | 0.770 | 0.143 |  | 0.868 | 0.078 |  | 0.015 |  | 0.063 |
| Unassigned | APEI3 | 0.999 | 0.135 |  | 0.332 | 0.062 |  | 0.584 | 0.159 |  |
|  | IFREMER | 0.447 | 0.882 |  | 0.354 | 0.975 |  | 0.730 | 0.620 |  |
|  | GSR | 0.554 | 0.416 |  | 0.930 | 0.404 |  | 0.696 | 0.428 |  |
|  | IOM.C |  |  | 0.500 |  |  | 1.000 |  |  | 0.500 |
|  | BGR.RA | 0.272 | 0.000 |  | 0.816 | 0.001 |  | 0.579 | 0.040 |  |
|  | BGR.PA | 0.455 | 0.033 |  | 0.506 | 0.031 |  | 0.545 | 0.110 |  |
| *Acantholaimus* | APEI3 | 0.365 | 0.212 |  | 0.913 | 0.008 |  | 0.753 | 0.007 |  |
|  | IFREMER | 0.235 | 0.005 |  | 0.020 |  | 0.063 | 0.115 | 0.001 |  |
|  | GSR | 0.489 | 0.022 |  | 0.794 | 0.006 |  | 0.412 | 0.012 |  |
|  | IOM.C |  |  | 1.000 |  |  | 1.000 |  |  | 1.000 |
|  | BGR.RA | 0.085 | 0.152 |  | 0.332 | 0.071 |  | 0.182 | 0.054 |  |
|  | BGR.PA | 0.365 | 0.071 |  | 0.244 | 0.014 |  | 0.134 | 0.038 |  |
| *Desmoscolex* | APEI3 |  |  | 0.500 |  |  | 1.000 |  |  | 0.500 |
|  | IFREMER | 0.364 | 0.833 |  | 0.107 | 0.701 |  | 0.959 | 0.436 |  |
|  | GSR | 0.215 | 0.616 |  | 0.862 | 0.827 |  | 0.245 | 0.408 |  |
|  | IOM.C |  |  | 0.500 |  |  | 0.500 |  |  | 1.000 |
|  | BGR.RA | 0.849 | 0.427 |  | 0.824 | 0.618 |  | 0.595 | 0.239 |  |
|  | BGR.PA | 0.536 | 0.466 |  | 0.494 | 0.194 |  | 0.471 | 0.501 |  |
| *Halalaimus* | APEI3 | 0.762 | 0.153 |  | 0.500 | 0.091 |  | 0.716 | 0.041 |  |
|  | IFREMER | 0.216 | 0.554 |  | 0.597 | 0.583 |  | 0.995 | 0.425 |  |
|  | GSR | 0.705 | 0.092 |  | 0.408 | 0.185 |  | 0.206 | 0.196 |  |
|  | IOM.C |  |  | 1.000 |  |  | 1.000 |  |  | 1.000 |
|  | BGR.RA | 0.866 | 0.170 |  | 0.729 | 0.488 |  | 0.356 | 0.125 |  |
|  | BGR.PA | 0.469 | 0.356 |  | 0.500 | 0.335 |  | 0.546 | 0.634 |  |

Table 12: Description of null models applied in the calculation of standard effect size (ses) of Phylogenetic Diversity (ses.PD), unweighted Mean Pairwise Distance (ses.MPD) and unweighted Mean Nearest Taxon Distance (ses.MNTD) for Nematoda, Genus-assigned, Unassigned, *Desmoscolex*, *Acantholaimus*, and *Halalaimus* ASVs in the BGR.PA, BGR.RA, IOM.C, GSR, IFREMER and APEI3 Areas. Source: R Package “picante” vignette.

| Null Model | Randomization |
| --- | --- |
| taxa.labels | Shuffle distance matrix labels (across all taxa included in distance matrix) |
| richness | Randomize community data matrix abundances within samples (maintains sample species richness) |
| frequency | Randomize community data matrix abundances within species (maintains species occurrence frequency) |
| sample.pool | Randomize community data matrix by drawing species from pool of species occurring  in at least one community (sample pool) with equal probability |
| phylogeny.pool | Randomize community data matrix by drawing species from pool of species occurring  in the distance matrix (phylogeny pool) with equal probability |
| independentswap | Randomize community data matrix with the independent swap algorithm^1^  maintaining species occurrence frequency and sample species richness |
| trialswap | Randomize community data matrix with the trial-swap algorithm^2^  maintaining species occurrence frequency and sample species richness |

**R scripts**

DADA2

<https://docs.google.com/document/d/11Mv0mmlyh-UNCwU8En5ceYC7RgoTPiqJh2tv2byadOs/edit?usp=sharing>

Downstream analysis

<https://docs.google.com/document/d/1iZB8hF4jfOzTuuBLWDB_qeZBPcNhhkvJMQ6KI1R7-nM/edit?usp=sharing>

References

1. Gotelli, N. J. Null model analysis of species co-occurrence patterns. *Ecology* **81**, 2606–2621 (2000).

2. Miklós, I. & Podani, J. Randomization of presence-absence matrices: Comments and new algorithms. *Ecology* **85**, 86–92 (2004).
